# Development of a delayed-release nutrient for appetite control in adults with obesity and type 2 diabetes and initial clinical testing in a single dose randomized controlled trial

**DOI:** 10.1038/s41387-019-0088-7

**Published:** 2019-07-15

**Authors:** E. Beale, E. Lim, H. Yassine, C. Azen, C. Christopher

**Affiliations:** 1 Division of Endocrinology and Diabetes, Keck School of Medicine, University of Southern California, Los Angeles, CA 90033 USA; 2 SC CTSI, Keck School of Medicine, University of Southern California, Los Angeles, CA 90033 USA; 3 CaliVive Inc., San Mateo, CA 94402 USA

**Keywords:** Drug development, Translational research

## Abstract

**Background and objectives:**

Delivery of nutrients directly to the small intestine, either via enteral feeding tube or by gastric bypass surgery, is associated with increased levels of appetite-suppressing and glucoregulatory hormones, including GLP-1, and reduced appetite. Achieving these changes non-invasively using formulated foods may be of therapeutic benefit in individuals with obesity and related comorbidities. The aim of this pilot study was to determine the effect of a single dose of a novel delayed-release nutrient (DRN) on glucose, GLP-1, c-peptide, insulin, and appetite in adults with obesity and type 2 diabetes.

**Subjects and methods:**

We formulated an all-natural, generally recognized as safe (‘GRAS”) DRN and conducted a randomized prospective crossover trial. Nineteen adults with obesity and type 2 diabetes underwent paired 3-h meal tolerance tests (MTT) in randomized order 1–4 weeks apart. Subjects ingested a single dose of DRN and the same nutrients as unformulated powders (UN).

**Results:**

For DRN compared with UN, the maximal concentration (Cmax) was significantly lower for glucose, c-peptide, and insulin, and the time of maximal concentration (Tmax) was significantly delayed. While Tmax for GLP-1 was also significantly delayed following DRN compared with UN (45 min later; *p* = 0.26), Cmax did not differ significantly. GLP-1 rose significantly during the last 90 min of the 3-h MTT (β_1_ = 0.16 pg/mL/min, *p* = 0.025), while following UN it decreased (β_1_ = −0.21 pg/mL/min, *p* = 0.0026) (*p* difference = 0.0003). There were minimal differences in seven measures of appetite and adverse symptoms between DRN and UN.

**Conclusions:**

We conclude that nutrient can be formulated using all-natural ingredients to induce a delayed rise in GLP-1. Further testing is needed to determine the amount and site of nutrient release, when maximum GLP-1 levels occur, and if modification of the formulation specifications and dose are associated with appetite and glucose control.

## Introduction

Increased distal nutrient delivery is now considered to initiate multiple gut-based neurohormonal pathways, including intestinal L-cell activation, that mediate the metabolic benefit of gastric bypass surgery^1–5^. This proposed key proximal mechanism is corroborated by numerous studies demonstrating that delivery of nutrient directly to the intestine via enteral feeding tube can acutely and significantly increase GLP-1 as well as other appetite-suppressing gut hormone levels, and increase satiety while reducing net caloric intake^6–9^.

We hypothesized that a formulation designed to release nutrient rapidly in the small intestine would simulate the appearance of nutrient in the small intestine, which occurs after gastric bypass surgery, and with nutrient administration by post-pyloric enteral feeding tube, and thereby increase GLP-1 release^10^. We propose that such a nutrient formulation could be ingested before, or even in place of, a regular meal to reduce appetite and caloric intake and improve glucose control.

As pharmaceutical materials used for delayed-release coatings of drugs and dietary supplements have limits on the amount that can be safely ingested, we investigated using natural polymeric carbohydrate coating materials. These coatings have the added benefit of meeting consumer preference for non-pharmaceutical therapies^11^. Currently, there are few reports of all-natural formulated nutrients for appetite and glucose control, and no commercially available products with demonstrated clinical efficacy^12–16^.

The aim of this pilot clinical trial was to evaluate in obese subjects with type 2 diabetes, the effects of ingestion of a single dose of a novel delayed-release nutrient formulation on circulating levels of GLP-1 and appetite compared with ingestion of the same amount of unformulated nutrient. We also evaluated the effects of this intervention on adverse symptoms and on levels of C-peptide, insulin, and glucose.

## Materials and methods

### Rationale for delayed-release nutrient specifications

The specifications for the delayed-release nutrient formulation were derived from multiple enteral feeding tube studies demonstrating that satiety and appetite regulating hormones are increased and total caloric intake is decreased through a variety of macronutrients delivered at different rates and sites in the small intestine, as summarized in a recent review by Alleleyn et al. (see Table 1)^8^. We have conducted studies using a mixed macronutrient formulation delivered by enteral feeding tube into the upper small intestine in adults with obesity and type 2 diabetes, and also in non-obese adults with type 1 diabetes. As a result, we identified a site, dose, mixed macronutrient composition, and rate of nutrient delivery that significantly enhanced levels of several glucose- and appetite- regulating hormones (GLP-1, PYY, and insulin), along with satiety^6^.

**Table 1:** Composition of administered delayed-release nutrient (DRN) and unformulated nutrient (UN)

	Delayed-release nutrient (DRN)	Unformulated nutrient (UN) and coatings
Energy (kcal)	200	200
Weight (g)	50	50
Coating materials weight (% weight gain)	15%	15%
Ethyl cellulose source	Surelease® Colorcon Harleysville, PA in commercially available coating Nutrateric® Nutritional Enteric Coating System, Colorcon Harleysville, PA	Dietary fiber cellulose, nutricology, alameda, CA
Ethyl cellulose (g)	5.9	5.9
Alginate source	NS Enteric® Colorcon Harleysville, PA in commercially available coating Nutrateric® Nutritional Enteric Coating System, Colorcon Harleysville, PA	Sodium alginate—food grade, distributed by Landor Enterprises, Inc. Williamsport, PA
Alginate (g)	0.7	0.7
Ratio ethyl cellulose: alginate	90:10	90:10
Sucrose source	SUGLETS® Sugar Spheres, Colorcon Harleysville, PA	SUGLETS^®^ Sugar Spheres, Colorcon Harleysville, PA
Sucrose spheres (g)	15	15
Sucrose spheres diameter (μm)	850–1000	850–1000
Sucrose spheres (kcal)	60	60
Whole milk powder source	Whole milk powder regular, Fonterra Co-operative Group Limited, Auckland, New Zealand	Whole milk powder regular, Fonterra Co-operative Group Limited, Auckland, New Zealand
Whole milk powder (g)	29	29
Whole milk powder-Carbohydrate (g)	11.6	11.6
Whole milk powder-Protein (g)	7.1	7.105
Whole milk powder-Fat (g)	7.6	7.627
Whole milk powder- (kcals)	145	145
Tonic water source	Schweppes Diet Tonic Water Dr. Pepper Snapple Group, Inc Plano, TX	Schweppes Diet Tonic Water Dr Pepper Snapple Group, Inc Plano, TX
Volume diet tonic water	11	11
Calories tonic water	0	0
pH tonic water	2.5	2.5

Based on our work and that of others, we aimed to develop a nutrient formulation that would allow delivery of ~200 kcal of nutrient several times a day for several months directly to the small intestine. Pharmaceutical delayed-release coatings have limits on the amount that can be safely ingested and prohibit their use in the quantities required when coating microparticles in the amounts of nutrient we desired to deliver. By contrast, there are no such limits on the small number of coatings composed of “generally recognized as safe” (“GRAS”) materials that have recently become commercially available^11,17,18^. The ethyl cellulose-alginate GRAS coating (Nutrateric^®^ Nutritional Enteric Coating System, Colorcon Harleysville, PA) was selected to provide enteric protection in the acidic pH of the stomach, while enabling release of nutrient in the alkaline upper intestine without concern for toxicity^19^. The size of the coated pellets was specified to be <2 mm to permit free passage across the pylorus^20^. The nutrient was ingested with non-caloric diet tonic water to maintain the integrity of the coating in the acidic stomach without addition of free nutrients to the ingested coated nutrient^21^.

The nutrients (sucrose and whole milk powder) and caloric load (200 kcal) used in the DRN formulation were selected to have a similar composition to those used in our previous studies using a mixed macronutrient liquid formulation (Ensure^®^ Original Nutrition Shake, Abbott) delivered by enteral feeding tube to the upper intestine ^6,7^.

### Manufacturing of DRN

The DRN was manufactured for the investigators at a commercial GMP manufacturing facility (Deseret Laboratories, Inc. St George, Utah). Commercially available sugar spheres (Suglets®, Colorcon, Harleysville, PA) were spray coated with whole milk powder to 200% weight gain. These particles were then coated with ethyl cellulose-alginate coating to 15% weight gain (Nutrateric^®^ Nutritional Enteric Coating System, Colorcon Harleysville, PA). Discrete pellets were manufactured such that 99.6% of particles had a sieve diameter of <2 mm.

### Dissolution Testing of DRN

**Fig. 1:**
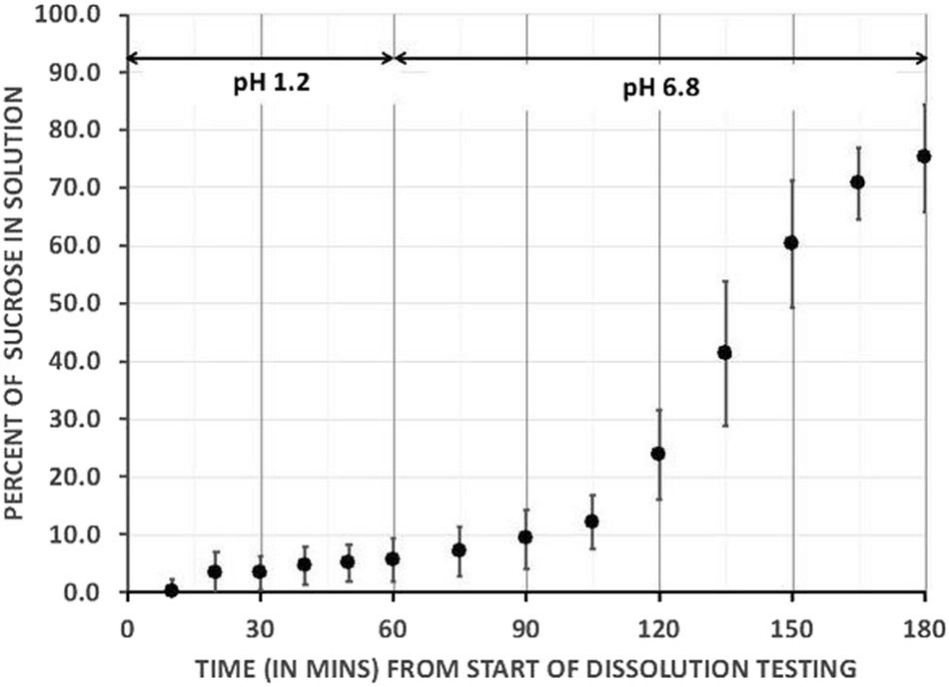
In vitro dissolution testing. The mean and standard deviation of three dissolution tests performed in vitro show significant increase in percent of sucrose in solution starting in minute 120–180 at pH 6.8

In vitro USP dissolution testing was conducted at 37 °C with paddle stirrers operating at 75 rpm with pH 1.2 (equivalent to empty gastric pH) for 1 h and then pH of 6.8 (equivalent to small intestinal pH) for a further 2 h (see Fig. 1). Sucrose release was measured on three dissolution tests as 6 ± 3.8% at 1 h, 24 ± 7.7% at 2 h, and 75 ± 9.3% at 3 h (Evolution 6100 Dissolution System).

### Study design

This was a prospective randomized crossover trial. Each subject underwent two 3-h meal tolerance tests (MTT) 1–4 weeks apart, once with coating applied to the macronutrient (DRN) and once with unformulated macronutrient, with powdered coating material (UN). As the coating material (Nutrateric^®^) was not available for ingestion as individual ingredients in powder form, commercially available substitutes in equivalent amounts to that used in the DRN were administered for the UN. The order of DRN and UN dosing was determined from a computer-generated randomization list generated by the PI (see Table 1). The research assistant enrolled the subjects and ascertained the order of treatment at the first study visit from sequentially numbered envelopes.

### Subjects

Key inclusion criteria were age 18–65 years, body mass index >27 kg/m^2^, type 2 diabetes with a duration <10 years and using only lifestyle modification, metformin, sulfonylureas, thiazolidinedione, or SGLT2 inhibitors for type 2 diabetes management. Key exclusion criteria were known foregut pathology or prior foregut surgery, previous surgical treatment for obesity and a history of pancreatitis. Use of insulin, DPP4 inhibitors or GLP-1 analog inhibitors in the previous 3 months and diet attempts using herbal supplements or over the counter medications in the past 1 month were also criteria for exclusion. Subjects with allergy to dairy products or intolerance to lactose were excluded from participation. The Western Institutional Review Board approved the study protocol. All subjects were screened for eligibility and provided written consent. The study statistician was blinded to the treatment group by use of group numbers in the dataset.

### Study visit preparation

All study visits took place at the University of Southern California Diabetes and Obesity Research Institute. Subjects fasted for 8 h from the evening before the start of the study visit, omitted their diabetes medications, and avoided vigorous exercise on the morning of their visit. Study visits were started between 8 and 10 am. Height, weight, and point of care HbA1c were measured at the first visit. Capillary blood glucose level was tested immediately prior to each MTT and was required to be between 70–200 mg/dL for the study to proceed.

### Study visit

Subjects reclined quietly on a phlebotomy chair at 45 degrees for the duration of the study without speaking or sleeping and were asked to read, use a computer, or watch non-food related television to occupy their time. They were permitted to get up to use the restroom. A peripheral intravenous line was placed with saline to keep the vein open. Two 4 mL blood samples were collected 5 min apart at −5 and 0 min to establish baseline. Immediately afterwards, the subjects were given 50 g (200 kcal) of DRN or UN, to ingest within 5 min with 235mls (an 11 fl. oz bottle) of room temperature diet tonic water. Study subjects were not blinded to the nutrient being administered but were informed only that any effects of the two nutrients on their appetite or blood results would be compared. DRN was spooned into the mouth and swallowed down with the diet tonic water without chewing on the pellets. UN was mixed with the diet tonic water to create a smooth drink. Blood samples were collected every 15 min for 3 h.

### Visual analog scale

At each blood draw, satiety, and adverse symptoms were assessed by the study subject using a visual analog scale^22–24^. Subjective feelings of hunger, fullness, satisfaction, and thoughts of food were recorded on a paper scale by marking a 150 mm line, without reference to previous responses. Nausea, abdominal pain, perspiration, and palpitations were also rated. (See Appendix A).

### Blood sample processing and assay

Samples were drawn into 4 ml EDTA vacutainers prepared with aprotinin (Phoenix Pharmaceuticals, Inc. Catalog #: RK-APRO): 1 mg/mL (500 KIU/mL) of blood and DPP4 inhibitor (Millipore, Catalog #: DPP4-010): 10 uL/mL of blood. After collection, samples were mixed by gentle inversion of the tube. The tube was then placed in an ice bath and centrifuged within 1 h of collection at 1500–2000 × *g* (RCF), for 15 min at 4 °C. Plasma (0.5 mls) was aliquoted into each of four cryovials. Cryovials were then immediately frozen and stored upright at −70 °C until assay. Batched assay was performed using a Millipore Human Metabolic Expanded Hormone Magnetic Bead Panel (Cat#HMHEMAG-34K-04, Insulin, C-peptide, GLP-1 (total). Plasma glucose concentrations were determined using an YSI 2300 autoanalyzer (Yellow Springs Instruments, Yellow Springs, OH).

### Data collection and statistical analysis

The specific aims of the study were to compare, in obese adult subjects with type 2 diabetes, levels of circulating GLP-1, C-peptide, insulin, glucose, satiety, and any adverse effects following DRN and UN in paired MTTs. The primary outcomes were maximum concentration (Cmax) and area under the curve (AUC) of total GLP-1. Secondary outcomes were Cmax and AUC levels of C-peptide, insulin, glucose, and the satiety and adverse effect scores on MTTs. Cmax is the average of each subject’s maximum level of GLP-1 following DRN and UN. Cmin and Tmin were reported for hunger and desire, since they decreased from baseline. In order to determine sample size we used data from two sources to derive mean changes in GLP-1 and the SD of the paired difference: (1) data from our group evaluating paired comparisons of three-hour enteral versus oral mixed meal tolerance tests in obese adults with type 2 diabetes^6^, and (2) data comparing pre-post gastric bypass in non-diabetics^25^. These data yielded a projected effect size of 0.68 (mean GLP-1 paired difference, divided by SD of difference); a sample of 20 participants was required to detect this effect with 80% power and testing at a 2-sided alpha of 0.05. The data were captured in a custom-designed REDCap database^26^. Data were screened, validated, corrected, and summarized prior to analysis for the number of observations, mean, standard deviation, minimum, and maximum for each variable. Random effects mixed models for 2-by-2 crossover designs were used to compare DRN and UN for Cmax, Tmax, AUC final-Initial value and the maximum rise over baseline. Exploratory analysis of slopes was performed by fitting linear regression models for the first and last 90-min periods for each subject. The resulting slopes were compared using random effects mixed models with interaction between period and time to compare paired differences between the first and the last 90 min within each treatment and between DRN and UN within each 90-min segment. Since this was conducted as a pilot study, no adjustment was made to *p*-values for the multiplicity of analyses. Statistical analyses were performed using SAS/STAT© version 9.2 software, at a 2-sided 0.05 significance level.

## Results

### Study flow and demographics

**Fig. 2:**
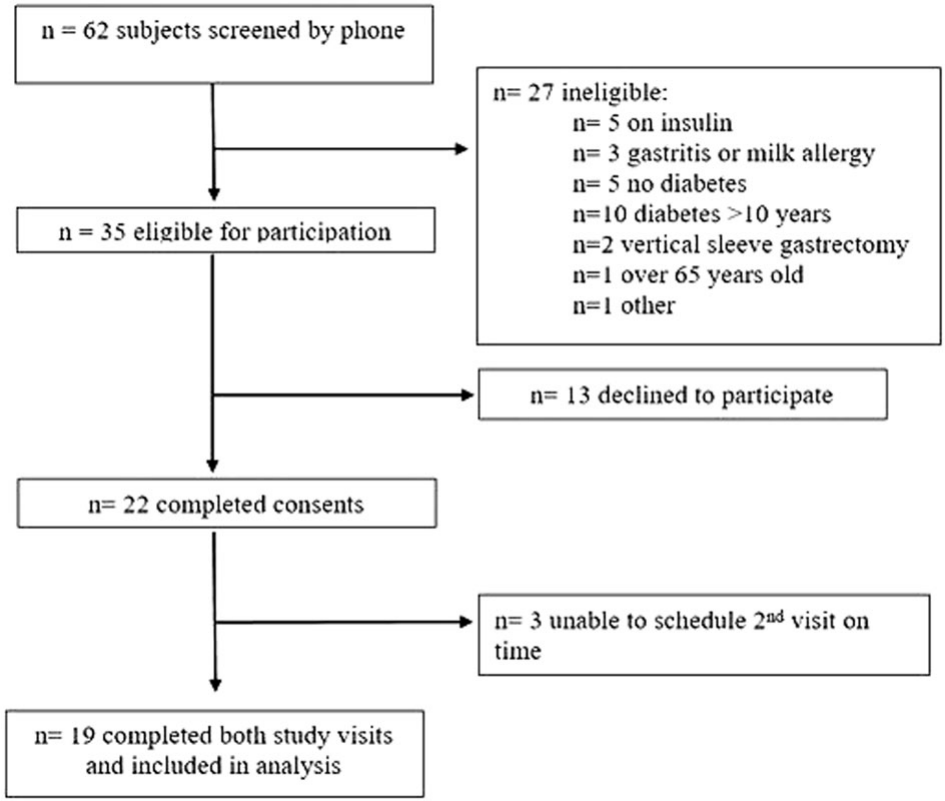
Subject flowchart. After screening 62 potential subjects by phone, 22 agreed to participate, and completed the consent process. However, due to scheduling difficulties, only a total of 19 subjects completed both study visits

Subjects were recruited from February to June 2017 and the study was conducted from March to July 2017. The flow of subjects through the study is shown in Fig. 2. Eighteen subjects were taking metformin, three pioglitazone and two a sulfonylurea. The average fasting capillary blood glucose at the start of each visit was 148.5 ± 34.3 mg/dL. Demographic data of the 19 subjects with paired MTTs included in analysis are shown in Table 2. The trial was completed after recruitment of target enrollment was reached.

**Table 2:** Demographics of subjects with paired meal tolerance tests (MTT)

Total subject (*N*)	19
Male: female	7:12
Age (years)	49 ± 8
Weight (kg)	106.7 ± 27.3
BMI (kg/m^2^)	38.7 ± 7.5
HbA1c (%)	7.4 ± 1.4
Duration with diagnosis of type 2 diabetes (years)	3.9 ± 2.5

### Hormone and glucose assays

The mean (SEM) and mean differences for DRN and UN, 95% confidence limits and p-values are shown for all hormone and glucose assays in Appendix B.

**Fig. 3:**
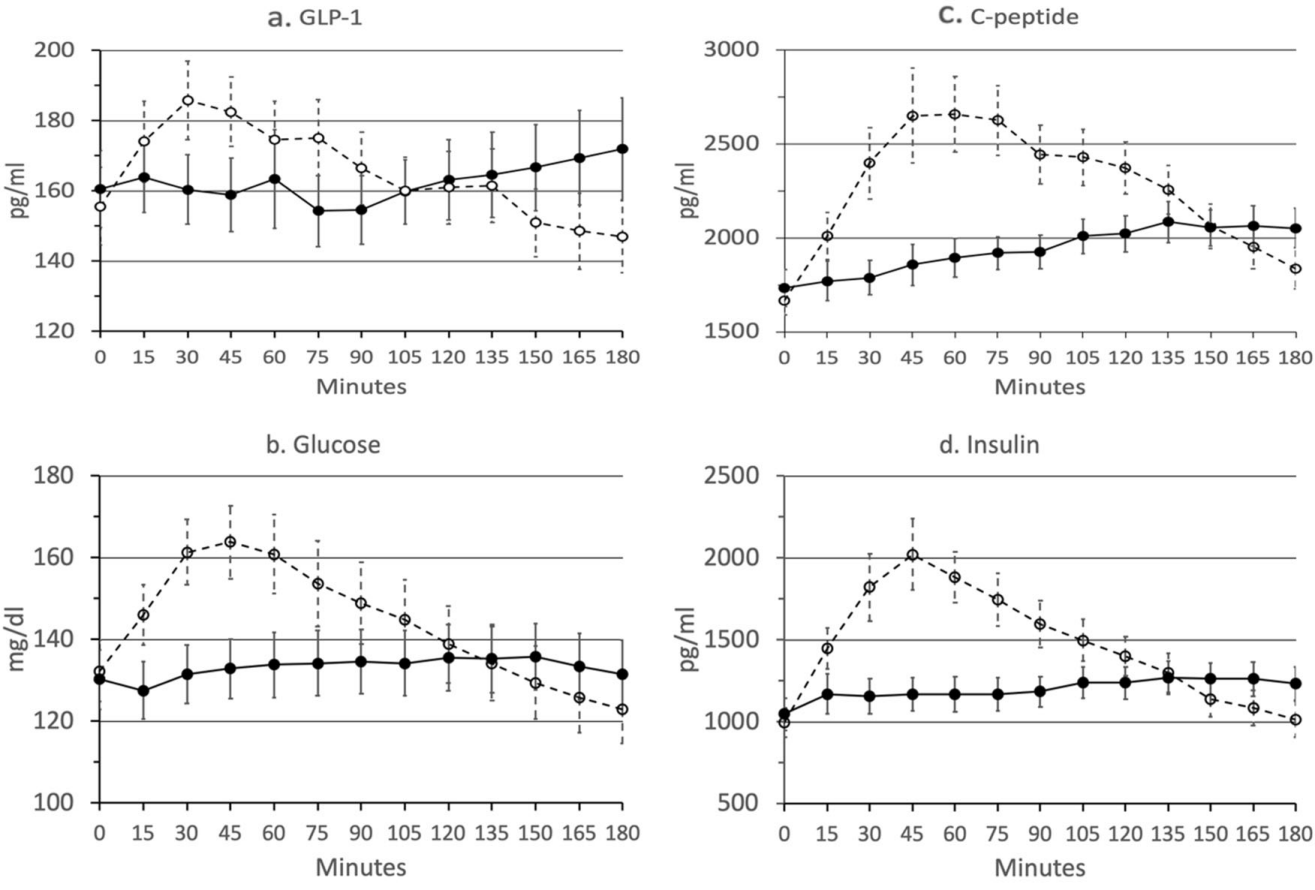
Average raw values for each analyte during 3-hour MTT (*n* = 19 pairs). Dotted line and open circles represent unformulated nutrient (UN). Solid Line and closed circles represent delayed-release nutrient (DRN). Data points represent mean (SEM) for all DRN and UN at each time point

Following UN, glucose and all hormones peaked in the first 90 min and this is not reflected in the slope, which is shallow for this period. Thus, only slope comparisons within the DRN group between the first and last 90 min, and between UN and DRN in the last 90 min are presented.

### GLP-1

GLP-1 Tmax occurred later following DRN than following UN (103 mins. vs. 58 mins., *p* = 0.03) and had a greater overall increase over baseline (11.6 pg/mL vs. −8.7 pg/mL, *p* = 0.02) (see Fig. 3a). However, Cmax GLP-1 levels, was not significantly different between DRN and UN and AUC for the study period was not was not significantly different between DRN and UN.

The slope of GLP-1 (pg/mL/min) increased during the last 90 min of the 3-h MTT following DRN (β_1_ = 0.16, *p* = 0.025), while it decreased following UN (β_1_ = −0.21, *p* = 0.003), and these slopes were significantly different (*p* = 0.0003) Within-group comparison of the slope between the first and last 90 min of the MTT was significant for DRN (*p* = 0.017).

### Glucose

Glucose Cmax was lower following DRN than following UN (142 mg/dL vs. 168 mg/dL, *p* < 0.0001) and Tmax later following DRN than following UN (84 min. vs. 43 min., *p* = 0.003), with a lower AUC (24 k vs. 26 k, *p* = 0.018), lower maximum rise over baseline (11.9 mg/dL vs. 35.3 mg/dL, *p* < 0.0001), and greater increase over baseline (1.10 mg/dL vs. −9.41 mg/dL, *p* = 0.0009) (see Fig. 3b).

Glucose slopes (mg/dL/min) for DRN was positive in the first 90 min (β_1_ = 0.07, *p* = 0. Slopes differed significantly between DRN and UN in the last 90 min (*p* < 0.0001). Within-group comparisons of slopes between first and last 90 min were significant for DRN (*p* = 0.0497).

### C-peptide

C-peptide Cmax was lower following DRN than following UN (2248 pg/mL vs. 2901 pg/mL, *p* = 0.0013) and Tmax later (130 mins. vs. 77 mins., *p* = 0.0003), with a lower AUC (350k vs. 414k, *p* < 0.0001) and lower maximum rise over baseline (518 pg/mL vs. 1235 pg/mL, *p* = 0.0002) (see Fig. 3c). The final rise over baseline was higher for DRN than UN but not significantly (319 pg/mL vs. 172 pg/mL, *p* = 0.08).

Slopes (pg/mL/min) for DRN were positive in the first 90 min (DRN β_1_ = 2.31 pg/mL/min, *p* = 0.035) and negative in the last 90 min for UN (β_1_ = −8.40, *p* < 0.0001). Slopes differed significantly between DRN and UN in last 90 min (*p* < 0.0001). Within-group comparisons of slope between the first and last 90 min were not significant for DRN (*p* = 0.26).

### Insulin

Insulin C-max was lower following DRN than following UN (1439 pg/mL vs. 2196 pg/mL, *p* = 0.0003) and Tmax was later following DRN than following UN (111 mins. vs. 59 mins, *p* < 0.0001), along with a lower AUC (216k vs. 269k, *p* = 0.0005), a lower maximum rise over baseline (395 pg/mL vs. 1201 pg/mL, *p* = 0.0001), and a greater final increase over baseline (187 pg/mL vs. 20 pg/mL, *p* = 0.0042) (see Fig. 3d).

Slopes (pg/mL/min) for UN negative in the last 90 min (β_1_ = −6.68, *p* < 0.0001), with no significant difference from zero in either period for DRN. Slopes differed significantly between DRN and UN in last 90 min (*p* < 0.0001). Within-group comparisons of slopes between first and last 90 min were not significant for DRN (*p* = 0.48).

### Satiety and adverse effects

Seven measures were evaluated at 14 time points at each MTT, with only two significant differences identified between DRN and UN. The lowest hunger score was earlier for DRN than UN (19 mins. vs. 44 mins, *p* = 0.0155). The maximum score for satisfaction was lower for DRN than UN (*p* = 0.0125). There was also a significant visit effect for satisfaction in that peak satisfaction occurred 28 min later at visit 1 than visit 2, regardless of treatment (DRN vs. UN).

## Discussion

Our overall aim is to formulate a nutrient that induces appetite and glucose control of therapeutic value via activation of a variety of neurohormonal intestinal pathways, including increased release of GLP-1 due to rapid release of nutrient in the upper small intestine. The formulation we developed met our particle size and in vitro pH and temporal release specifications. However, the effects on appetite and gut hormones were not similar to those seen following enteral feeding tube studies for uncertain reasons. There was, however, a delayed rise in GLP-1 with formulated nutrient, with levels still rising at the end of the 3-h study window.

Studies using an enteral feed tube to administer different doses of nutrient to the intestine support our strategy of using delayed-release nutrient to induce appetite and glucose control^6–8^. We previously demonstrated that 250 kcals of mixed nutrient delivered directly to the upper small intestine by feeding tube rapidly induces a rise in GLP-1 and satiety^6^. To administer a similar amount of nutrient in an orally ingested formulated version without concern for toxicity we used a GRAS coating. By contrast Ma et al. administered two doses of lauric acid with a far lower caloric load (22.5 kcal) coated in methacrylic acid—methyl methacrylate copolymer, a non-GRAS coating (Eudragit L100; Evonik Industries AG, Darmstadt, Germany) at two sequential meals^13^. The authors demonstrated that GLP-1 was higher and glucose lower after the second dose of coated nutrient compared with placebo. Effects on appetite were not reported. The dose of coating material used by Ma et al. was at the upper approved limit for daily intake, which could limit long-term use.

In the recent publication by Alleleyn et al.^16^, all-natural particles of similar size and nutrient composition to those used in the current study but with a modified whey coating were formulated with the goal of increasing nutrient delivery to the distal ileum in order to activate the ileal brake. A small but significant increase in satiety and decrease in hunger was seen by 15 min after ingestion compared with a control nutrient and similarly there was a there was a small reduction in caloric intake of a subsequent ad libitum meal.

In the current study, unformulated nutrient showed the expected significant rise in glucose and hormones over the first hour and then fall to baseline by the end of the 3-h study. By contrast, maximal levels following the delayed-release nutrient occurred later, and were lower, except for GLP-1, which continued to rise at 3 h. Furthermore, measures of hormones, with delayed-release nutrient for glucose and appetite were lower than those seen following delivery of a similar caloric load to the upper intestine by enteral feeding tube and gastric bypass ^6–8,25^.

Given that we did not directly measure when, where, and how much nutrient was released we do not know why appetite and hormonal changes differed from those hypothesized based on in vitro testing. We propose several possible reasons for our findings.

Firstly, the 3-h meal tolerance test might have been too short to allow for maximum nutrient release and effect on GLP-1, appetite, and other outcome measures. The GRAS nutrient formulation used in the clinical study had in vitro dissolution characteristics suggesting that the majority of the nutrient load would be released in vivo in the small intestine within 3 h of ingestion. However, non-sucrose nutrient release from the particles may not have occurred at the rate observed in the in vitro dissolution testing, which measured only sucrose release. Morphological changes may have occurred in the formulated nutrient in the gastrointestinal tract that altered the coating, or core nutrient release. For example, swelling of ethyl cellulose may have prevented pH-dependent alginate pore formation and nutrient release.

Secondly, gastric emptying may have been slower than expected. We hypothesized that the 2-mm particles would pass through the stomach at the rate of plain water^20^. The particles may, however, have sedimented in the stomach of the semi-recumbent subjects^27^. It is also possible that as nutrient, gastric acid, and quinine from the tonic water were released into the intestine, they slowed gastric emptying delaying intestinal delivery of the full caloric load^28,29^. This slowing of nutrient delivery is not present when nutrient is administered by enteral feeding tube or following gastric bypass^30,31^.

Some formulated nutrient may have passed through unabsorbed to the large intestine. In this case symptoms such as bloating, or diarrhea may have occurred. However, no such symptoms were reported by the subjects.

A limitation of this study is that the site, length, and rate of nutrient exposure, that determine hormonal and appetite effect of nutrient, were not assessed^32^. In vitro gastrointestinal simulation testing is not readily available, is poorly standardized, and correlation with human testing weak^33^. All methods have several drawbacks including radiation exposure, limited temporal resolution, and failure to distinguish between various gastrointestinal segments^34^. Labeling the active ingredient with drugs or radioisotopes is not permitted at food (GMP) manufacturing facilities. We did not add coating markers of gut transit and absorption after production as there was no evidence that the marker would transit the intestine with the nutrient particles. Lactulose as used in hydrogen breath testing may accelerate small bowel transit. Furthermore, marker coatings might alter nutrient release from the particles and could also be hazardous to the research team and subjects. Iterative in vitro formulation modification and in vivo testing was considered to be the most pragmatic developmental strategy. An ad libitum meal is a feasible means of assessing any effect on total caloric intake ^16^.

The tonic water used to ingest the nutrients contains quinine may have altered gut hormone release and appetite^35,36^. However, as the same solution was used with both meals differences between the groups are unlikely to be due to the quinine.

We assume that several macronutrients stimulated GLP-1 release, and that both nutrient and GLP-1 stimulated c-peptide release. This might explain why peak insulinemia with UN corresponds to a blood glucose peak rather than follows it as might be expected following glucose administration. While GLP-1 levels rise late in the study following DRN administration this is not associated with a rise in insulin. This may be because threshold levels of GLP-1, nutrient and glycemia needed to stimulate insulin release were not achieved.

With respect to limitations of the statistical analysis, the large number of statistical comparisons could be considered a limitation in interpretation of results. However, where there was a significant difference between modes, the *p*-values tended to be very small, so testing at a smaller significance level, e.g., *p* < 0.01 or even 0.005 would only affect three results: Glucose AUC, GLP-1 Tmax, and GLP-1 final-initial would no longer be significant.

This work provides additional information regarding the use of formulated nutrient for appetite and glucose control. The formulation we evaluated was comprised of ingredients that are widely consumed. The dietary fiber coating may provide health benefits^37–39^. The findings of this study may be generalizable to other obese adults with type 2 diabetes. It is possible that the study subjects who had type 2 diabetes could have had altered gastrointestinal motility that could have affected findings and that would not be expected to be present in obese adults without diabetes.

Numerous areas in the field of targeted enteral nutrient require further study. Testing this formulation for a longer period and with multiple doses in ambulatory subjects would provide a more complete picture of the GLP-1 profile and effects on appetite. The formulation may be modified to provide a higher caloric load and different nutrient cores and coatings. Future studies will adjust the experimental design to focus on outcome measures for either appetite and caloric intake, or glycemic control, rather than both.

## Conclusion

This study explores the use of formulated nutrient to control appetite and dysglycemia. It adds to the limited published research by presenting data on the use of a novel delayed-release natural coating to deliver nutrient directly to the intestine in obese adults with type 2 diabetes. Findings demonstrate that GLP-1 release can be delayed with this strategy, but further work is needed to develop an optimal formulation that achieves the desired effects on gut hormones and appetite. We have identified several areas for further research including options for modifying the nutrient formula, and the development of more widely available and feasible methods of assessing gastrointestinal transit and nutrient absorption in humans.

## Appendix A

**Details of visual analog score**

Subjects were asked “How hungry do you feel?”, “How full do you feel?”, “How satisfied do you feel?” and “How much do you think you can eat now?”. The scales were anchored at either end by statements; “I am not hungry at all/I am not full at all/I am completely empty/nothing at all” on the left and “I am as hungry as I have ever been/I am totally full/I cannot eat another bite/a large amount” on the right. Adverse symptom questions were anchored at either end by statements; “I have no nausea/abdominal pain/perspiration/palpitations at all” on the left and “I have a lot of nausea/ abdominal pain/perspiration/palpitations” on the right.

## Appendix B

Intervention means and mean differences for delayed-relase (DRN) vs. unformulated nutrient (UN)

**Table:**

Measure	DRN: Mean (SE)	UN: Mean (SE)	Mean difference: DRN minus UN (SE)	95% confidence interval	*p*-value
GLP-1 (pg/mL)					
Cmax	192.89 (12.75)	194.88 (12.75)	−1.99 (10.75)	(−24.68, 20.70)	0.86
Tmax (minutes)	103.23 (13.17)	57.82 (13.17)	45.42 (18.64)	(6.08, 84.75)	0.026
AUC	29177 (1872.38)	29884 (1872.38)	−706.39 (1085.50)	(−2996.60, 1583.81)	0.52
Maximum rise over Baseline	32.53 (6.78)	39.06 (6.78)	−6.52 (9.59)	(−26.76, 13.71)	0.51
Final–initial	11.55 (5.69)	−8.71 (5.69)	20.26 (7.88)	(3.63, 36.89)	0.02
Glucose (mg/dL)					
Cmax	142.20 (8.49)	167.61 (8.49)	−25.41 (5.01)	(−35.98, −14.84)	<0.0001
Tmax (minutes)	84.01 (9.0)	43.09 (9.0)	40.92 (11.85)	(15.91, 65.92)	0.003
AUC	23997 (1489.44)	26021 (1489.44)	−2024.18 (775.18)	(−3659.66, −388.69)	0.018
Maximum rise over Baseline	11.94 (2.68)	35.34 (2.68)	−23.40 (3.10)	(−29.94, −16.86)	<0.0001
Final–initial	1.10 (3.07)	−9.41 (3.07)	10.51 (3.63)	(4.96, 16.05)	0.0009
Insulin (pg/mL)					
Cmax	1439.09 (162.84)	2195.78 (162.84)	−756.69 (167.20)	(−1109.45, −403.93)	0.0003
Tmax (minutes)	111.10 (6.64)	59.43 (8.64)	51.67 (9.92)	(30.74, 72.60)	<0.0001
AUC	216417 (19563)	268827 (19563)	−52410 (12316)	(−78395, −26425)	0.0005
Maximum rise over Baseline	394.77 (141.31)	1201.28 (141.31)	−806.51 (161.34)	(−1146.91, −466.11)	0.0001
Final–initial	186.80 (38.52)	19.87 (38.52)	166.93 (50.57)	(60.25, 273.62)	0.0042
C-Peptide (pg/mL)					
Cmax	2248.24 (185.17)	2901.81 (185.17)	−653.57 (170.23)	(−1012.71, −294.42)	0.0013
Tmax (minutes)	129.84 (9.23)	77.04 (9.23)	52.83 (11.66)	(28.23, 77.43)	0.0003
AUC	349525 (21166)	413522 (21166)	−63997 (11926)	(−89159, −38835)	<0.0001
Maximum rise over baseline	517.76 (147.05)	1235.28 (147.05)	−717.52 (151.96)	(−1038.12, −396.92)	0.0002
Final–initial	318.97 (61.47)	171.83 (61.47)	147.14 (79.32)	(−20.21, 314.49)	0.08

*P*-values from linear mixed effects model for crossover design
